# Failure of Intravenous Lipid Emulsion to Reduce Diazinon-induced Acute Toxicity: a Pilot Study in Rats 

**Published:** 2013

**Authors:** Mohammad Moshiri, Maryam Vahabzadeh, Leila Etemad, Hossein Hosseinzadeh

**Affiliations:** a *Department of Pharmacodynamy and Toxicology, School of Pharmacy, Mashhad University of Medical Sciences, Mashhad, Iran.*; b *Pharmaceutical Research Center, Mashhad University of Medical Sciences, Mashhad, Iran.*; c *Pharmaceutical Research Center, Department of Pharmacodynamy and Toxicology, School of Pharmacy, Mashhad University of Medical Sciences, Mashhad, Iran. *

**Keywords:** Diazinon, Intravenous lipid emulsion, Organophosphorus

## Abstract

Diazinon (DZN) is a synthetic organophosphorus (OPs) insecticide widely used in agricultural and household applications. OPs, particularly DZN, are highly lipid soluble liquids. Intravenous lipid emulsion (ILE) has been shown to reduce toxicity caused by some lipid soluble agents. We evaluated the antidote effect of ILE on acute toxicity of DZN. Twenty-four Sprague-Dawley female rats weighting 200-250 g were treated orally with dose of 480 mg/ kg of DZN gavaged at the volume of 0.5 mL/kg. Thirty minutes after administration of DZN, two groups were treated by either ILE 10% (ILE10) or normal saline (NS) (16 mL/kg) (NS16) that were infused for the duration of 15 minutes. Another two groups were also treated by either ILE 20% (ILE20) or NS (10 mL/kg: NS10) as above. The changes in body weight, diarrhea score, muscular power, fasciculation, convulsions and mortality rate of the animals were all monitored immediately after infusions and then every 6 h up to 48 h. There was no significant difference in animals mean weight between different groups during the observation period. In addition, during the 48-hour observation we could not find any difference in muscular power and diarrhea score between groups of ILE20-NS10 and ILE10-NS16 in comparison with each other, and neither ILE 10% nor ILE %20 could not reduce mortality rate of animals or increase the survival time of rats. In conclusion, ILE seems to be unable to reverse DZN acute toxicity and it might be due to conversion of DZN to potent and less lipid soluble agent.

## Introduction

Intravenous lipid emulsion (ILE) (intralipid) has been shown to reduce toxicity caused by some lipid soluble drugs (1). In many studies ILE has been an effective antidote in various types of intoxicated animals by different groups of drugs such as local anesthetics (2, 3), propranolol (4) and tricyclic antidepressants (5, 6) Furthermore, ILE has been able to decrease morbidity and mortality in some intoxicated patients (7, 8). However, it was not completely effective in some poisoning such as clomipramine and paroxon (9-11). 

Diazinon (DZN) is a synthetic organophosphorus (OPs) insecticide widely used in agricultural, commercial, and household applications. Diazinon was classified as a class II ‘moderately hazardous’ pesticide by The World Health Organization (WHO) (12). There are approximately 3 million pesticide poisonings world-wide resulting in 220,000 deaths every year (13) and pesticide poisons are one of the main causes of poisonings (14, 15), or poison-related death in Iran (16). Diazinon as other OPs, act by interfering with nervous system function (17) Atropine and oximes are antidotes against acute toxicity of DZN (12) and some material such as saffron have antidotal effect against its sub acute toxicity (18, 19). OPs, particularly DZN, are highly lipid soluble liquids (20). DZN is hydrolyzed by water and some products which are more toxic than DZN itself (21). Some products of soybean oil are used as stabilizers, react with water preventing the hydrolysis of DZN, and subsequently form products that are more toxic. This mechanism, which has been also used by manufacturers, reduces DZN toxicity and enhances its median lethal dose (LD50) (21).

We evaluated the antidotal effect of ILE (intralipid) on toxicity of organophosphate DZN, which is considered as a lipid-soluble substance.

## Experimental

*Animals and preparations:*

Twenty-four non-pregnant Sprague-Dawley female rats weighting 200-250 g were housed in cages with a 12 h light/dark cycle and easy access to food and water. Animals were divided into four groups of consisting six rats. 

Since DZN (BazodinR, Cyngenta, purity 96%) is poorly water soluble, it was dissolved in corn oil (Sigma) to gavage to animal. We also used ILE 10% (Lipovenoes® 10% , Germany ) or ILE20% (intralipid emulsion [Fresenius Kabi AB] Spain) or normal saline (NS) for intravenous treatment .

*Evaluation of ILE on DZN toxicity*

All of the animals were gavaged with dose of 480 mg/kg of DZN which was dissolved in corn oil at the gavaged volume of 0.5 mL/kg. Rats were food-deprived overnight before the experiment in order to reduce the possible interaction between food and the toxin.

Appropriate rodent restrainers were used to limit animal movements. The devices had been washed before each trial in order to prevent pheromonal-induced stress or cross-infection. Subsequently, a 25G plastic vein cannula was inserted in lateral tail vein under aseptic condition and attached to the animal tail properly.

Thirty minutes after ingesting DZN, two groups were treated by either ILE 10% (ILE10) or Normal saline (16 mL/kg) (NS16) that were infused for the duration of 15 min. Another two groups were also treated by either ILE 20% or NS (10 mL/kg) (ILE 20 and NS 10 groups) which were infused during 15 min. By the end of infusions, (ILE or NS) animals were placed in separate cages with easy access to food and water.

The changes in body weight, diarrhea score, muscular power, presence of fasciculation, and convulsions and mortality rate of the animals were all monitored immediately after infusions and then every 6 hours up to 48 h. Diarrhea was scored as follow: 0 = normal; 1 = loose stool; and 3 = defecation incontinence (22). The muscular powers of animals were evaluated using modified De Bleecker scoring- Grade 4: normal mobility; Grade 3: ataxic gait; Grade 2: stretch movements after tail stimulation; Grade 1: standing after tail stimulation; Grade 0: no voluntary movements after tail stimulation, four limb paralysis (23). All animals were evaluated by an individual unaware of the groups.

*Statistics*

Results are expressed as mean ± standard deviations and were analyzed with T non-paired test and Mann–Whitney test by using Spss11.5 software. Statistical significance was set at P < 0.05 for all tests.

## Results

At the beginning of the experiment, there was no significant difference in animals mean weight between different groups and it either showed insignificant changes during the further observation (Figurs 1 and 2). In addition, during the 48 h observation we could not find any difference in muscular power between groups of ILE20-NS10 and ILE10 - NS16 in comparison with each other (Figure 3 and 4). 

**Figure 1:**
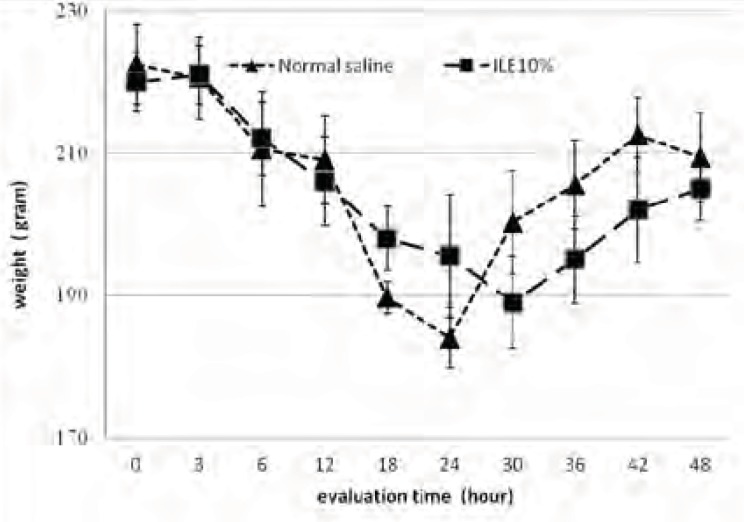
Effect of ILE 10% (16 mL/kg intravenous lipid emulsion 10%) in compare NS16 (16 mL/kg normal saline on means of weight of rats which were intoxicated by diazion. P=non-significant. numbers of animals in each group at 0 h = 6 and at 48 h = 2

**Figure 2:**
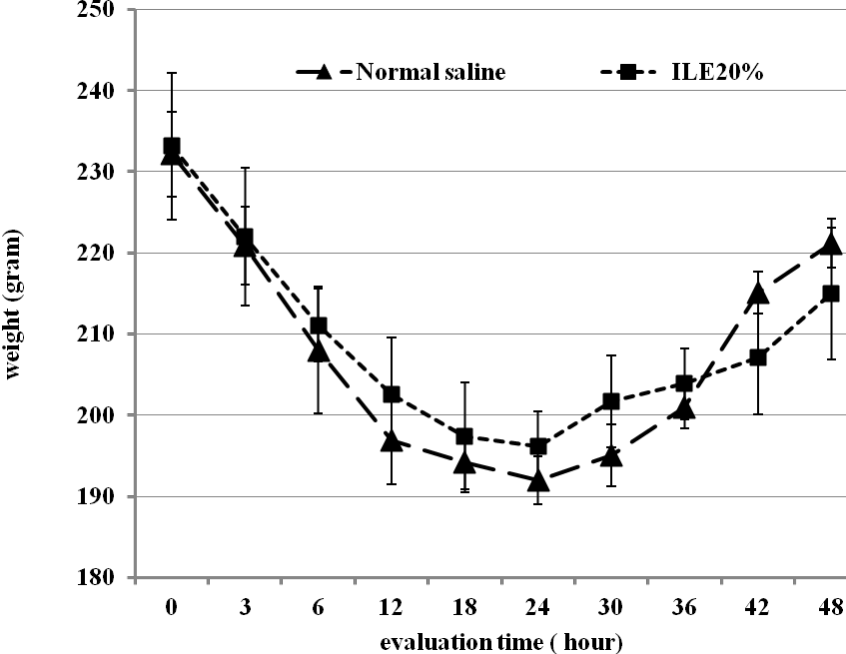
Effect of ILE 20% (10 mL/kg intravenous lipid emulsion 20%) in compare NS10 (10 mL/ kg normal saline on means of weight of rats which were intoxicated by diazion. P= Non-significant. numbers of animals in each group at 0 h = 6 and at 48 h = 2

**Figure 3:**
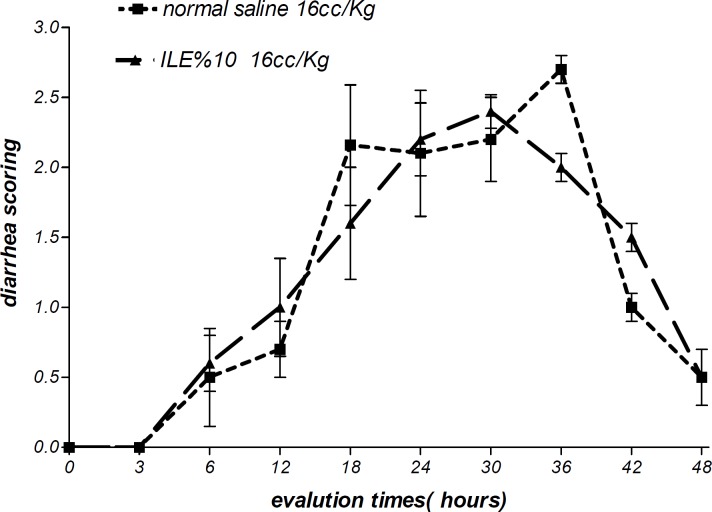
Effect of ILE 10% (16 mL/kg intravenous lipid emulsion 10%) in compare NS16 (16 mL/kg normal saline) on means of muscle power score of rats which were intoxicated by diazion. P= Non-significant. numbers of animals in each group at 0 h = 6 and at 48 h =2

**Figure 4:**
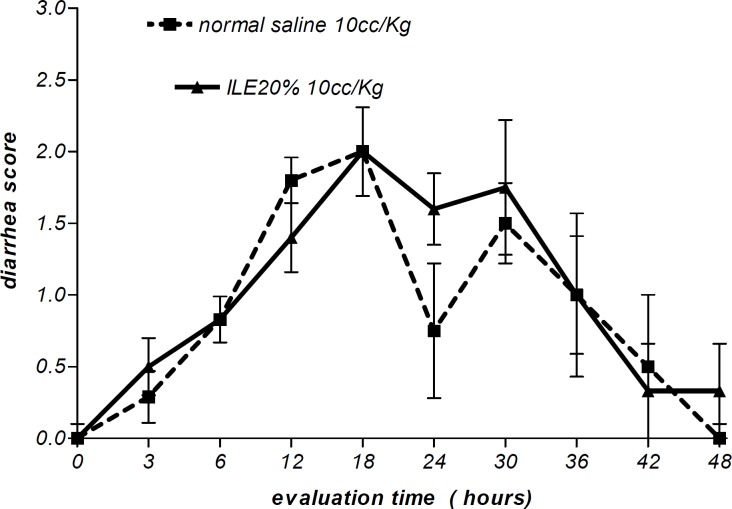
Effect of ILE 20% (10 mL/kg intravenous lipid emulsion 20%) in compare NS10 (10 mL/kg normal saline) on means of muscle power score of rats which were intoxicated by diazion. P= Non-significant. numbers of animals in each group at 0 h = 6 and at 48 h = 2

**Figure 5:**
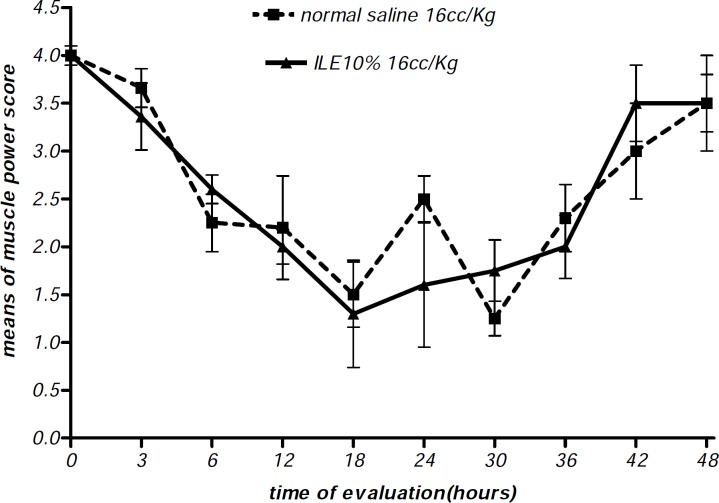
Effect of ILE 10% (16 mL/kg intravenous lipid emulsion 10%) in compare NS16 (16 mL/ kg normal saline) on means of diarrhea score of rats which were intoxicated by diazion. P= Non-significant. numbers of animals in each group at 0 h = 6 and at 48 h = 2.

**Figure 6:**
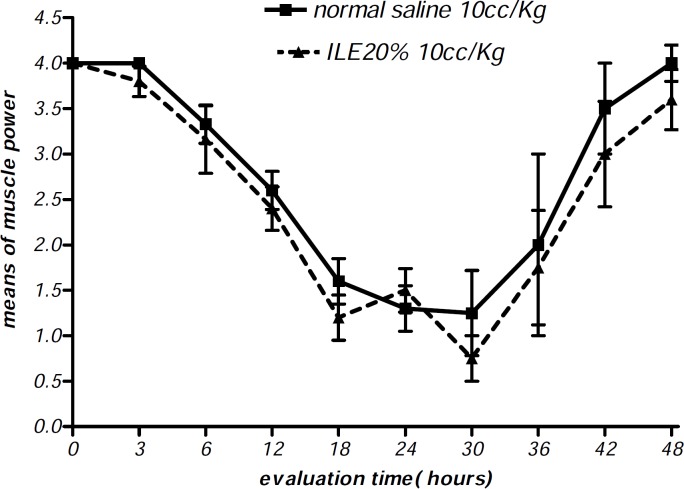
Effect of ILE 20% (10 ml/kg intravenous lipid emulsion 20%) in compare NS10 (10 ml/kg normal saline) on means of diarrhea score of rats which were intoxicated by diazion. P= Non-significant. numbers of animals in each group at 0 h = 6 and at 48 h = 2

Moreover, there were no alterations in diarrhea score among all animal groups. Thus, comparing to normal saline, both concentrations of ILE seemed to be unable to reduce diarrhea score in poisoned animals (Figures 5 and 6) and neither ILE 10% nor ILE %20 could not reduce the mortality rate of animals or increase the survival time of rats (Table 1).

The incidence of fasciculation between four groups was insignificant during different times of observation. Convulsion did not occur in any of the animals. 

**Table 1:** comparing of mortality rate and means of survival times of four groups

	**normal saline 16cc**	**ILE 10%**	**p-VALUE**	**normal saline 10cc**	**ILE 20%**		**p-VALUE**
means of survival time (hours)	33.0 ± 5.3	34.0 ± 6.1	0.91 (NS)	35.0 ± 5.9		37.0 ± 6.8	0.83 (NS)
mortality rate	66.67%	66.67%	1.00 (NS)	66.67%		50.00%	1.00 (NS)

ILE10% = intravenous lipid emulsion 10%, ILE20% = intravenous lipid emulsion 20%, numbers of animals in each group at 0 h = 6

## Discussion

Since 2006 when the first case was reported to be rescued by ILE (24), ILE has been evaluated as antidote in several kinds of drugs toxicity, such as local anesthetics, tricyclic antidepressants (1, 10, 25), propranolol (4), atenolol (26), and numerous other compounds. All compounds, which ILE has decreased their toxicity, have high lipid-solubility properties. The most important mechanism of action which has been suggested for antidote effect of ILE is “Lipid sink theory” (27-29). Based on such theory, ILE acts as a as a new compartment in blood that lipophilic substances can be drawn into the “lipid sink”, resulting in a concentration gradient between tissue and blood. This phenomenon causes toxin or drug to distance from the target tissues into the “lipid sink” of ILE (30). 

OPs and especially DZN are extremely lipid-soluble toxins (31), and according to the “lipid sink” theory and the hydrolysis-inhibitory effect of ILE (21), it is supposed to reduce the toxicity of DZN. Nevertheless, we failed to confirm this hypothesis, likewise the research by Bania on the effect of ILE on another OP, Paraoxon (11). Both concentrations of ILE (10% and 20%) failed to either reduce mortality rate or increase the survival time in comparison with normal saline.

OPs inhibit acetylcholinesterase (AChE), the critical and widespread nervous system enzyme (31, 32), which degrades the neurotransmitter acetylcholine into choline and acetic acid, resulting in overstimulation of muscarinic and nicotinic receptors (20). The muscarinic overstimulation could induce muscarinic sings (diarrhea, over secretion of exocrine glands), and nicotinic overstimulation could induce nicotinic signs (fasciculation, convulsion and muscles paralysis). We evaluated the body-weight changes as an indicator of water loss due to diarrhea and over secretion of exocrine glands, which had dehydrated the animals. Neither muscarinic nor nicotinic signs of DZN toxicity were improved by different concentration of ILE.

The three main classes of OPs insecticides are phosphorothionates, phosphorodithioates, and phosphoroamidothiolates (33, 34). DZN belongs to phosphorothionate OPs, which are weak inhibitors of AChE (33, 35). Whilst phosphorothionate OPs undergo metabolic activation (desulfatation) to their corresponding oxygen analogues (oxon), they become extremely more potent (100-folds) (33, 35, 36). The oxon product of DZN, diazoxon, has a high affinity and potency to phosphorylate the serine hydroxyl group within the active site of AChE (33). Some authors believe that DZN do not directly inhibits AChE and must first be metabolized to diazoxon (33, 37). DZN and other OPs rapidly absorb following oral administration (32) and undergo a high hepatic first pass metabolism (33) as only 35% of oral dose will be eventually bioavailable (33). The activation of DZN to diazoxon is mediated by cytochrome P_450 _primarily within the liver, although some extrahepatic metabolism, such as the brain, has been reported (33, 38) Diazoxon is not as much lipophilic as its parent substance, DZN, (39). So it has 10 times less affinity for lipid comparing with DZN (40). The Partition coefficients (n-octanol-water) of Diazoxon and DZN are 2.07 and 3.81 respectedly (41). Thus, diazoxon is less trapped in fat compartments than DZN (42). Therefore, the major part of DZN changes into a 1000-times more potent and 10-times less lipid soluble product, diazoxon, prior to access to blood circulation.

AS mentioned above the most important mechanism for antidotal effect of ILE is the “Lipid Sink” mechanism (29, 43) ,which an intravascular lipid compartment would be formed while adding a large amount of lipids into the blood. Such repartitioning can distant xenobiotics from the site of toxicity into the blood and send them to the liver to be metabolized and detoxified (44, 45).

In conclusion, ILE seems to be unable to reverse DZN acute toxicity, and it might be due to conversion of DZN to potent and less lipid soluble agent. 
